# Coexisting Phases of Individual VO_2_ Nanoparticles for Multilevel Nanoscale Memory

**DOI:** 10.1021/acsnano.4c13188

**Published:** 2025-01-02

**Authors:** Peter Kepič, Michal Horák, Jiří Kabát, Martin Hájek, Andrea Konečná, Tomáš Šikola, Filip Ligmajer

**Affiliations:** † Brno University of Technology, Central European Institute of Technology, Purkyňova 123, 612 00 Brno, Czech Republic; ‡ Institute of Physical Engineering, Brno University of Technology, Faculty of Mechanical Engineering, Technická 2, 616 69 Brno, Czech Republic

**Keywords:** vanadium dioxide, phase-change memory, nanophotonics, transmission electron microscopy, insulator−metal transition, coexisting phases, hysteresis

## Abstract

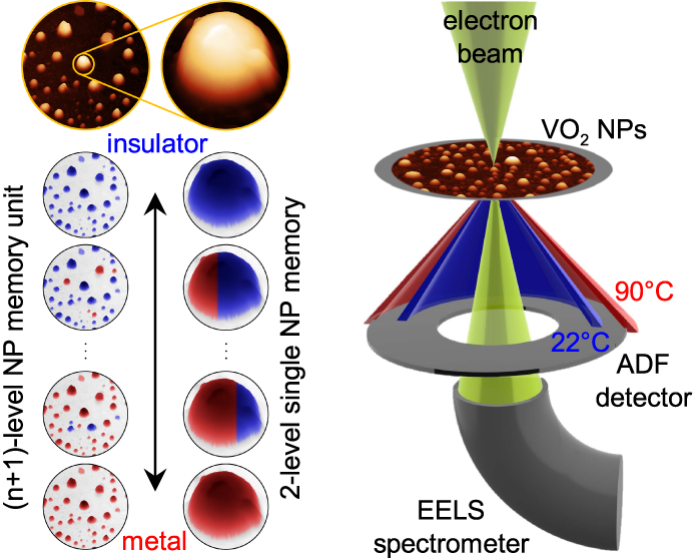

Vanadium dioxide (VO_2_) has received significant interest in the context of nanophotonic metamaterials and memories owing to its reversible insulator–metal transition associated with significant changes in its optical and electronic properties. The phase transition of VO_2_ has been extensively studied for several decades, and the ways how to control its hysteresis characteristics relevant for memory applications have significantly improved. However, the hysteresis dynamics and stability of coexisting phases during the transition have not been studied on the level of individual single-crystal VO_2_ nanoparticles (NPs), although they represent the fundamental component of ordinary polycrystalline films and can also act like nanoscale memory units on their own. Here, employing transmission electron microscopy techniques, we investigate phase transitions of single VO_2_ NPs in real time. Our analysis reveals the statistical distribution of the transition temperature and steepness and how they differ during forward (heating) and backward (cooling) transitions. We evaluate the stability of coexisting phases in individual NPs and prove the persistent multilevel memory at near room temperatures using only a few VO_2_ NPs. Our findings unveil the physical mechanisms that govern the hysteresis of VO_2_ at the nanoscale and establish VO_2_ NPs as a promising component of optoelectronic and memory devices with enhanced functionalities.

## Introduction

**Figure 1:**
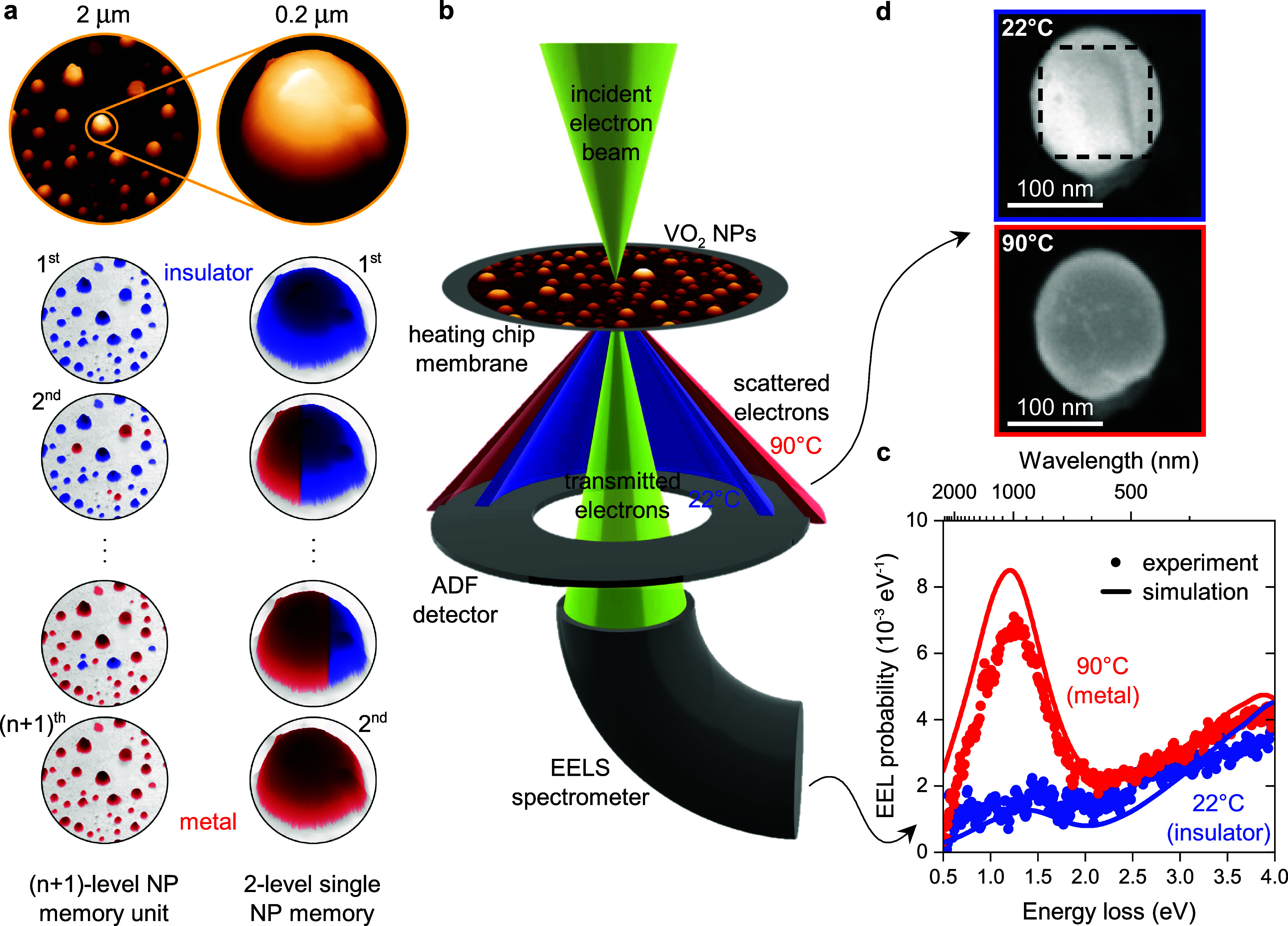
VO_2_ NP memory and the measurement setup. (a) Concept of a VO_2_ NP memory unit based on coexisting insulator (blue) and metal (red) phases within a single VO_2_ NP. The left column corresponds to (*n* + 1)-level memory based on an *n*-NP ensemble, and the right column corresponds to a 2-level single-NP memory. The illustration uses real atomic force microscopy (AFM) images of our samples. (b) Scheme of EEL spectroscopy and ADF measurement of VO_2_ NPs at different temperatures. (c) Measured and simulated EEL spectra of a 130 nm VO_2_ NP on a SiN membrane in the insulator (22 °C; blue) and metal (90 °C; red) phases. (d) ADF images of the NP in (c) at the listed temperatures. The dashed rectangle highlights the area over which EEL spectra in (c) were spatially integrated.

Phase-change materials, with their solid–solid transition accompanied by a significant change of the optical properties, play an important role in the rapidly developing field of nanophotonics. Tunable metasurfaces for active control of light waves^1^ or integrated photonic memories for novel types of computing and data storage^2^ represent key areas of these materials applications. On-and-off states of the optical memories are represented by high and low optical transmission, which is controlled by materials that have multiple phases with distinct optical properties.^3^ The most utilized materials in this field are chalcogenide glasses, typically germanium antimony telluride (GST) compounds, as they exhibit a nonvolatile amorphous–crystalline phase transition accompanied by significant optical modulation.^4^ For example, a patch of GST on top of a waveguide can store several levels of optically readable information and retain them for decades.^5^ Unfortunately, while energy consumption for retaining the data is zero, writing them (amorphizing the material) requires an ultrafast melt-quench process at above 600 °C and 10 °C/ns.^6^ This process can be very energy-consuming, especially when only volatile memory is sufficient, as in neuromorphic computing,^7^ random access memories,^8^ or reconfigurable metasurfaces.^9^ In such scenarios, a volatile phase-change material, vanadium dioxide (VO_2_), represents a more efficient solution because its insulator–metal transition (IMT, from monoclinic to rutile phase) is available at around 68 °C and at microscale requires only nanojoules of energy.^10−12^ Due to the inherent phase-change hysteresis, the VO_2_’s metal–insulator transition (MIT) in the reverse direction (cooling) occurs at lower temperatures, around 60 °C. Single-crystal VO_2_ nanoparticles (NPs), with their MIT reaching 30 °C,^13,14^ could lower the retention energy even more by bringing the whole platform closer to the room temperature. Moreover, they could increase the bit compactness (the number of optical levels per area) because the exact transition temperature and absorption of each (lithographically) separated NP (see Figure 1a) is easier to control than that of a film where the transition is more stochastic,^15^ and the absorption is averaged out. The coexistence of phases, already demonstrated for single-crystal films^16,17^ and nanobeams,^18,19^ brings another degree of freedom that can be exploited at the NP level for multilevel memories. So far, it has been shown that the hysteresis characteristics relevant for the memory applications (transition temperature, steepness, and contrast) can be controlled by stoichiometry, substrate-induced strain, volume, defects and doping.^13,14,20−25^ For example, one can decrease the transition temperature by tungsten dopants, or increase transition steepness via strain from lattice-mismatched substrate. However, these characteristics have been studied and controlled in VO_2_ films,^20−22^ and ensembles of VO_2_ NPs,^13,14,23−25^ but not in the individual single-crystal VO_2_ NPs, which represent the smallest unit (monocrystalline grains) of the polycrystalline films and large nanostructures. As those hysteresis characteristics of films and nanostructures represent only the average value of their grains, it is then difficult to fully control them, especially when they are integrated into photonic devices. Therefore, the understanding of the individual single-crystal VO_2_ NP phase transition and the low-temperature stability of its coexisting phases is important from both the fundamental and the application point of view. Regarding applications, the possible coexistence of the insulating and metallic phases, observed so far only in microscale nanobeams,^26,27^ could also bring another degree of freedom, elevating the number of VO_2_ memory levels.

Here, we report on our study of individual single-crystal VO_2_ NPs by scanning transmission electron microscopy (STEM) techniques, which provide superior spatial resolution and correlation of high-resolution images with the spectroscopic information.^28,29^ While we thermally induced the phase transition, we correlated the evolution of electron energy loss (EEL) spectra with annular dark-field (ADF) structural contrast (see Figure 1b). Thus, we have been able to recognize the individual coexisting phases and analyze their spatial distribution and hysteretic behavior within individual VO_2_ NPs in real time. By investigating the hystereses of hundreds of NPs, we have found that the macroscale VO_2_ IMT consists of gradual transitions with transition temperatures that are narrowly spread, while the MIT is formed by extremely abrupt transitions at temperatures that are spread across a larger temperature window. Ultimately, we examined the stability of the coexisting phases at lower temperatures for both individual NPs and NP ensembles. Based on these findings, we demonstrate an optical memory unit in the form of a VO_2_ NP ensemble, where the number of memory levels is related to the number of NPs (see Figure 1a). These results indicate additional possible counterparts to the already established VO_2_ optical memory devices,^11,12^ and strengthen the position of VO_2_ as an optical unit for information storage and processing.

## Results and Discussion

### Probing the Phase Transition by STEM

To verify the possibility of probing the phase transitions of individual VO_2_ NPs by STEM, we fabricated them by dewetting a VO_2_ film (see Methods) on a commercial SiN membrane on a heating chip (Protochips Inc., USA). The dewetting process was chosen because it provides well-separated single-crystal hemispherical NPs (see images and size distribution in Figure S1), with low MIT temperatures even on top of amorphous substrates.^13,14^

Figure 1c shows the measured and simulated EEL spectra of a VO_2_ NP (diameter 130 nm), below and above the transition temperature. A prominent plasmonic peak, emerging at 1.24 eV in the spectrum of the metallic phase (further described in Figure S2), clearly confirms the IMT. Investigating such transitions in other NPs (see Figure S3a), we found that the EEL contrast (the signal difference between the insulator and metal phase) linearly increases with the NP diameter. As this trend relates to the increasing optical absorption of NPs (see Figure S3b), the results from the EEL measurement can be directly linked to the far-field optical properties of VO_2_ NPs.

When looking at the NP image obtained by the ADF detector (Figure 1d), we can see a distinct change in the contrast upon the IMT. The ADF detector records elastically scattered electrons at a specific angular range.^30^ The ADF contrast thus can be related to either the physical movement of the NP or the structural transformation during the transition of the NP.^31^ In a recent comprehensive study, we confirmed that the ADF signal can be directly linked to the change of crystal structure associated with the phase transition.^32^ Using various TEM and STEM techniques we characterized lattice and electronic signatures of the phase transition, and we ruled out all the other possibilities of artifacts and cross-talks that could be caused by electron beam-induced surface oxidation or other chemical and geometrical size modifications. Namely, there we provide direct evidence of the structural change associated with the change of ADF contrast by means of high-resolution TEM (related to atomic structure), electron diffraction (reciprocal space structure), low-loss EELS (electronic structure), and core-loss EELS (chemical and electronic structure). With the established correlation of phase-change and ADF contrast, we can extend our analysis toward real-time phase transition dynamics at the level of individual NPs, which would be otherwise hardly accessible using far-field optical measurements.

### Hysteresis of Individual VO_2_ NPs

**Figure 2:**
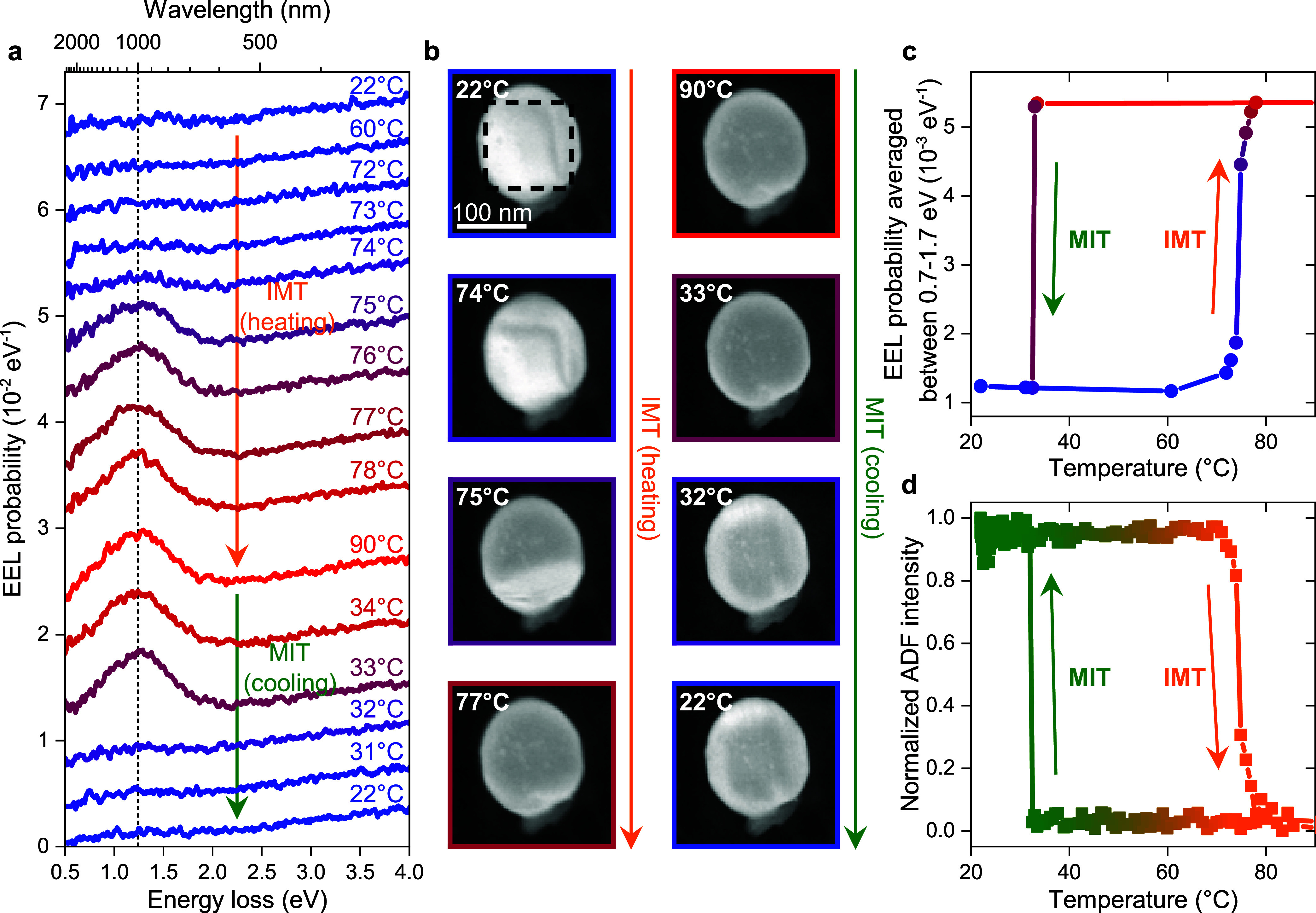
Phase transition hysteresis of a single VO_2_ NP. (a) EEL spectra of a single VO_2_ NP recorded at temperatures listed in the graph, as the NP is driven through its IMT and back. The spectra are stacked by 0.004 eV^–1^ shifts. The vertical dashed line highlights the position of the plasmon resonance, and the arrows indicate the direction of the IMT (orange, heating) and MIT (green, cooling). (b) ADF images of the NP at the listed temperatures. The dashed rectangle highlights the area over which EEL spectra in (a) were spatially integrated. (c) Phase transition hysteresis extracted from the EEL spectra in (a), averaged between 0.7–1.7 eV. (d) Phase transition hysteresis extracted from the mean ADF intensity of the NP in (b).

The EEL spectra and ADF images of the NP investigated in Figure 1c,d were taken sequentially during the phase transition to observe its dynamics. In Figure 2a, we can see the temperature evolution of the EEL spectra during the heating and cooling cycle. Upon extracting the EEL probability (averaged between 0.7–1.7 eV) and plotting it as a function of temperature, we can observe the single-NP hysteresis (Figure 2c). The gradual IMT around 74.5 °C indicates the presence of coexisting phases (also confirmed in Figure S4). The abrupt MIT at 32.7 °C, on the other hand, indicates an avalanche-like transition from a supercooled state.^33,34^ Note that such a transition does not exclude the presence of coexisting phases during the MIT, but it makes their observation harder: the activation volume (the minimum size of an initial nucleus) for the MIT is often greater than that for the IMT,^35^ and can be even greater than the volume of the investigated NP.^36^ Note that we observed this phase coexistence also in other NPs (see Figure S5b). Interestingly, the phase coexistence was also visible in ADF images, as shown in Figure 2b, where the NP gets darker upon the IMT. By processing the sequence of ADF images (see Methods), we were able to reconstruct (Figure 2d) the same hysteresis of the NP as with the EEL data. In general, the ADF approach was three times faster and delivered a hundred times lower electron dose to the sample than the EEL measurement. This fact is especially important when it is necessary to avoid the spurious influence of the high-energy electrons on the sample (see Figure S5). The ADF technique thus facilitates localized analysis of structural transitions and has the potential to examine other phase-change materials at the nanoscale or to disentangle the mechanism of nonvolatile ramp-reversal memory behavior of VO_2_.^37,38^ In our case, it allowed us to analyze the phase transition hysteresis of hundreds of VO_2_ NPs simultaneously and to discover important differences between IMT and MIT, as we will describe now.

### Statistics of VO_2_ Hysteresis Properties

**Figure 3:**
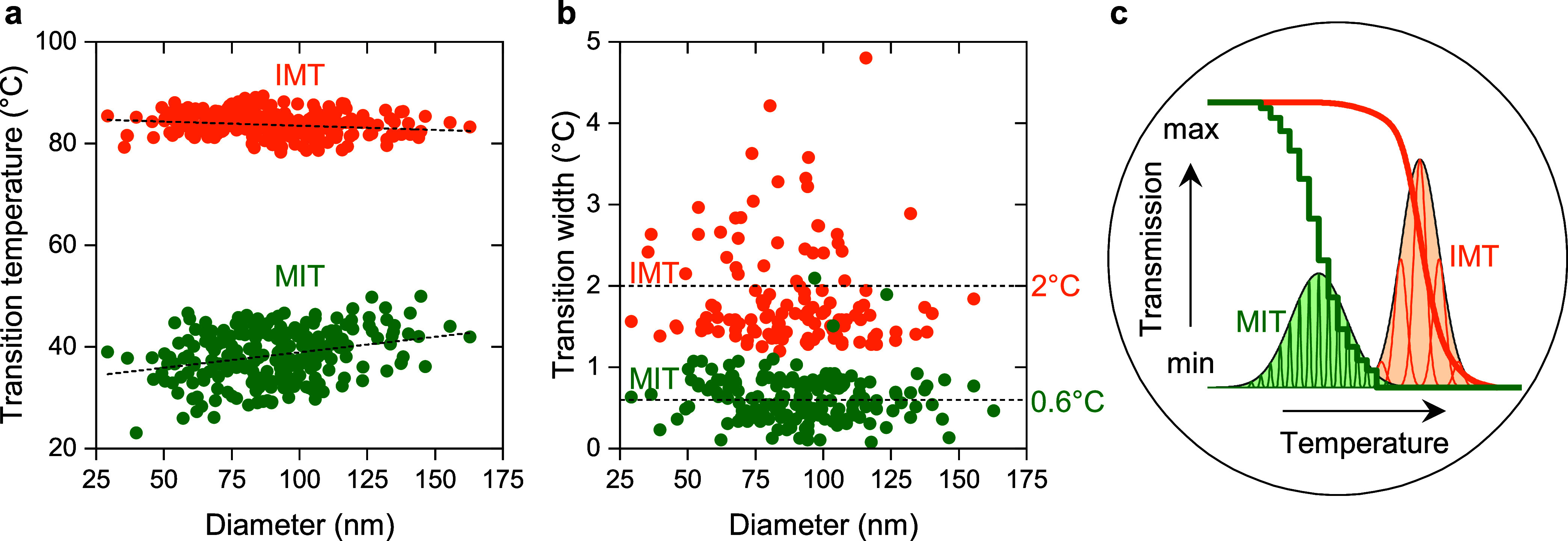
Statistics of the phase transition hysteresis of VO_2_ NPs. (a) Size-dependent distribution of transition temperatures and (b) transition widths of the IMT (orange) and MIT (green) of various NPs. Dashed lines represent linear fits and averaged values in (a) and (b), respectively. (c) Schematic of the phase transition hysteresis of a polycrystalline VO_2_ film, which we model as a system of individual NPs. Each NP is represented by a peak, with the peak widths and positions reflecting the statistical distributions obtained in panels (a) and (b). The overall behavior (e.g., transmission) of the polycrystalline film is then an aggregate of transitions of the individual grains (modeled by NPs).

In most polycrystalline VO_2_ films and nanostructures, the steepness of the IMT is practically identical to that of MIT.^24,39−41^ There are, however, films where these two transitions differ significantly.^42,43^ To better understand the shape of these large-scale transitions from the single-grain perspective, we recorded video-rate sequences of ADF images (2 fps, 1 °C/s, see Video 1) and utilized them to simultaneously analyze phase transition hystereses of hundreds of VO_2_ NPs. The extracted size dependence of transition temperatures during IMT (orange) and MIT (green) of 266 VO_2_ NPs is plotted in Figure 3a. Although the IMT temperature is weakly dependent on the diameter of a NP (decreases by 0.02 °C/nm), the MIT temperature is three times more sensitive to increasing diameter (increases by 0.06 °C/nm). The hysteresis broadening at low NP volumes observed here and by others^13,25^ thus seems to be predominantly related to the MIT. Moreover, the statistical variation across all NPs, which arises from the local inhomogeneities within individual NPs^18^ and the inherent stochasticity of the transitions,^15,38^ is more than twice as wide for MIT temperatures (Δ*T* ≈ 24 °C) compared to IMT temperatures (Δ*T* ≈ 11 °C).

We also analyzed the transition widths of our NPs (the temperature range required to completely switch a NP from one phase to another) and report the results in Figure 3b: While we cannot see any clear size dependence of the IMT and MIT transition widths, we find that the average IMT width (2.0 °C) is more than three times larger than the average MIT width (0.6 °C). Note that such a narrow MIT width can even be an overestimation, as it was limited by the chosen 0.5 °C step and by the curve-fitting process (see Methods). Also, this observation does not exclude the possibility of size-dependent transition widths for larger NPs or NPs with different shapes. Despite the very abrupt nature of the MIT in the vast majority of NPs, three of them (1%) exhibited coexisting phases during the MIT nevertheless (see the green data points above 1 °C in Figures 3b and S6). Our findings confirm that the coexistence of insulating and metallic phases in VO_2_ of microscale volumes^18,19^ is also present at the previously unexplored limit of nanoscale volumes. Moreover, coexisting phases at the nanoscale are at least 70 times less probable during the MIT than during the IMT (3 NPs versus 210 NPs). The observed difference in the transition widths indirectly confirms that the activation volume during the MIT (discussed in the previous section) is larger than during the IMT. In the context of a hypothetical multilevel phase-coexistence memory based on VO_2_ NPs, the retention temperature can thus be set right above the abrupt MIT, which is near the ambient temperature.

Based on these statistical findings, we put forward a model for the phase transition of polycrystalline VO_2_ films and microstructures, where the size of the grains is generally similar to the sizes of NPs investigated here. So far, the phase transition has been modeled based on the macroscopic properties of such films or on the aggregated properties of discrete NP ensembles.^13,14,22^ In our description, the macroscopic forward transition is composed of the IMTs of individual grains, which have broad transition widths, but their transition temperatures have a very narrow spread around the central value (Figure 3c). During backward MIT, on the other hand, the transitions of the grains are very abrupt (narrow MIT widths) but spread over a larger temperature window (Δ*T*), which we justify in our model by inequalities of grain boundaries^24^ and strain^18^ in a polycrystalline film. These findings provide valuable insights into the physics of VO_2_ hysteresis and coexisting phases within NPs, which are important for designing optical memories. In the following paragraphs, we will focus more on the concept of a multilevel optical memory based on VO_2_ NPs and further investigate the low-temperature stability and hysteresis of the individual NP’s coexisting phases.

### Multilevel Optical Memory

**Figure 4:**
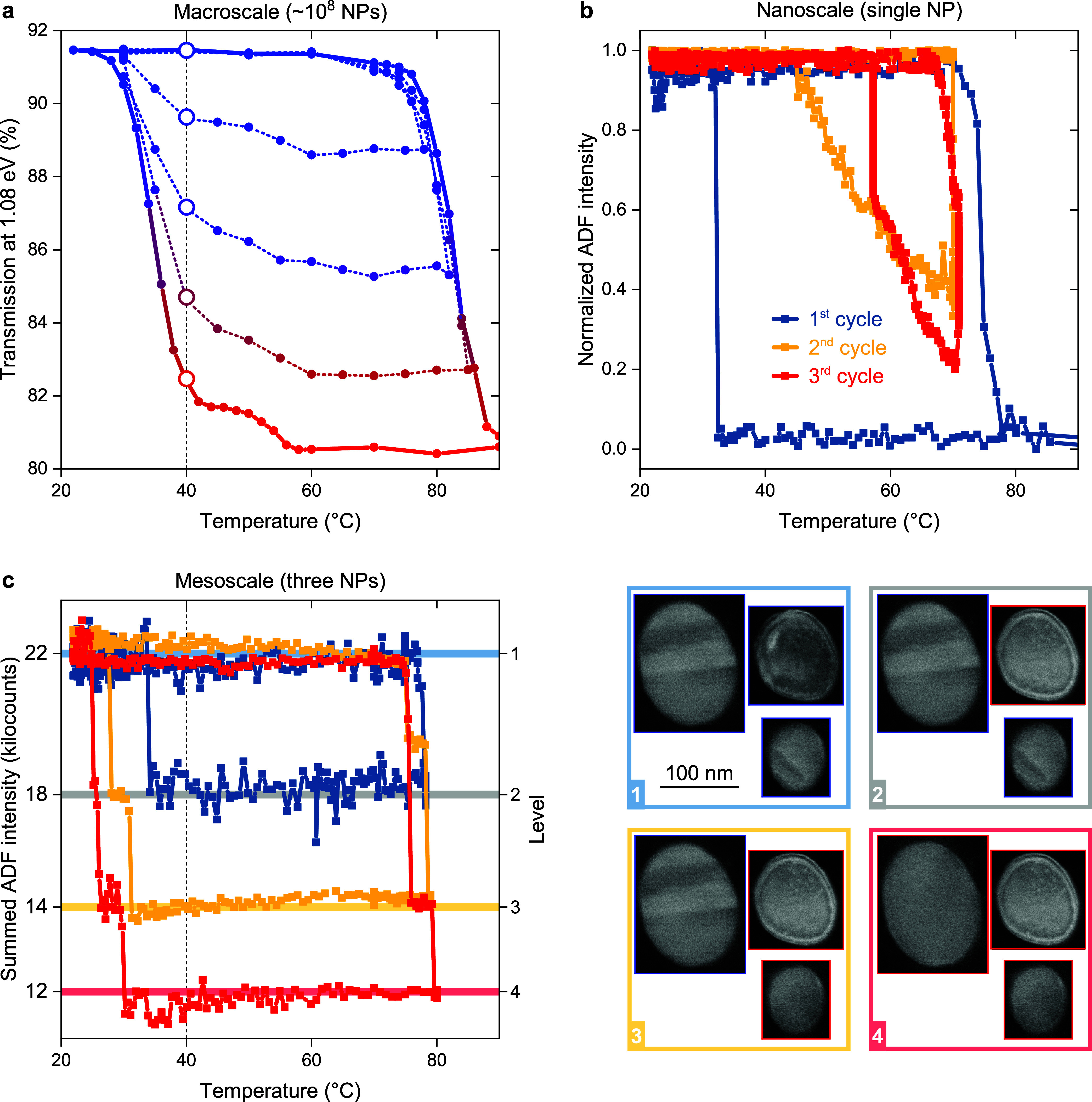
Stability of coexisting phases and a mesoscale optical VO_2_ memory unit. (a) Phase transition hysteresis of a macroscale ensemble of VO_2_ NPs extracted from their far-field transmission at 1.08 eV, with one full and three partial heating–cooling cycles. A multilevel far-field optical memory can be realized with such an ensemble at a temperature as low as 40 °C (vertical dashed line). Note the memory levels are well separated from each other, beyond the noise observable at the outermost hysteresis. (b) Stability of the coexisting phases within a single nanoscale VO_2_ NP, examined during one full and two partial heating–cooling cycles using normalized ADF intensity. (c) Summed ADF intensity of three VO_2_ NPs (a mesoscale memory concept) as they experience three heating–cooling cycles. The data indicate that such an ensemble can represent a 4-level mesoscale memory stable at 40 °C. Micrographs next to the graph show ADF images of the studied NPs during those three cycles, with blue and red rectangles highlighting the insulating and metallic phases, respectively. Note that NP images are artificially assembled to be next to each other compared to the original positions in the video.

Already in 2015, Lei et al.^26^ demonstrated a persistent memory effect in a hybrid system composed of a VO_2_ substrate and plasmonic nanoantennas. In their work, multiple memory levels of optical extinction spanning the full range of the hysteresis loop were optically addressed when the hybrid system was kept at different starting temperatures during the IMT. Analogically, we kept the VO_2_ NPs in various mixtures of phases by terminating the IMT at distinct temperatures. This allowed us to create a macroscale free-space optical memory device (Figure 4a) that possesses multiple levels of transmission states even at a convenient temperature of 40 °C. However, to reduce the footprint and scale down a potential memory device, it is essential to understand if the intermediate transmission levels are formed by a mixture of fully insulating and fully metallic NPs or by NPs that are switched to different degrees, i.e., with coexisting phases.

Our ADF imaging strategy allowed us to investigate the stability and coexistence of VO_2_ phases within individual NPs. We focused on the NP already shown in Figure 2, which exhibited such a coexistence and applied three heating–cooling cycles with the heating terminated at three distinct temperatures to stabilize three levels of the phase coexistence (Figure 4b). In the first cycle, the full IMT was completed, and the heating terminated at the temperature of 90 °C. The NP then retained its metallic state upon cooling by more than 50 °C down to 32 °C. In the second cooling cycle, when the heating terminated at 70 °C while the NP exhibited a phase coexistence (with approximately 65% of the metallic phase), the NP was continuously switching back to the insulating state instead of partially retaining the metallic state, and the MIT was completed already at 45 °C. In the third cycle, when we terminated the heating at 71 °C (with approximately 80% metallic phase) and cooled the system down to 57 °C, we even observed spontaneous propagation of the insulating phase at constant temperature by approximately 0.2 nm/s until the NP was fully switched. Based on these observations, also confirmed by several other NPs (see Video 2), we infer that coexisting phases within single VO_2_ NPs are not stable at lower temperatures, which means that there are no hysteresis subloops analogical to those of macroscopic NP ensembles (Figure 4a). However, the slowly moving phase front does not preclude the coexistence of phases at shorter time scales. To identify the true limits of coexisting phases within the MIT of VO_2_ NPs for memory and neuromorphic computing applications,^7^ a rigorous ultrafast study is needed, analogously to studies done on VO_2_ films, single-crystalline nanobeams and NP ensembles.^16,18,44^ We envision that analogous changes in image contrast can help resolve this question when used in an ultrafast pump–probe TEM setup.^45^ Until then, the unstable metallic phase of the individual NP at low temperatures conclusively confirms that the intermediate transmission levels of large-scale devices (such as in Figure 4a) are formed by a mixture of fully insulating and fully metallic NPs (as proposed in ref (13) and recently indicated in ref (14)).

Although a macroscopic ensemble of VO_2_ NPs can be used as a persistent multilevel memory, the low stability of coexisting phases in single individual VO_2_ NPs prevents their use in nanoscale long-term multilevel memory applications. These two extreme cases imply that an intermediate design must exist corresponding to a mesoscale ensemble of VO_2_ NPs that can retain a delocalized intermediate state with the lowest possible footprint. We, therefore, experimentally analyzed the phase transition hysteresis of an ensemble of three VO_2_ NPs using the sum of their ADF intensities as a metric. We chose those particular NPs because their footprint can be kept below 500 nm × 500 nm and because they exhibited sequential, cumulative switching during at least three recorded cycles (see Video 2). In Figure 4c, we can see the sum of the ADF intensity of the three VO_2_ NPs. During the first cycle, where heating stopped at 78 °C, only one NP went through the IMT while maintaining its metallic phase until 34 °C. During the second cycle (stopped at 79 °C), also the second NP was switched, albeit before the first NP. This inconsistency with the first cycle, which arises from the statistical distribution of the IMT temperatures, must be properly addressed when the actual integrated optical memory is designed. The reverse MIT during the second cycle occurred below 31 °C for both NPs. During the third cycle (stopped at 80 °C), also the last NP finally switched to the metallic state, and all NPs returned to their insulating states when cooled below 30 °C. Note that the MIT temperature of the first two NPs decreased after each cycle. Such a sequential decrease most likely results from the accumulated electron dose from the electron beam (as confirmed by our analysis reported in Figure S7), which was shown to drive the NPs to ultimately retain the metallic state even at room temperature (Figure S5). Note that although unwanted during analysis like this one, such an effect can be useful for postfabrication trimming of VO_2_ hysteresis. On the other hand, this behavior should not be an issue in the photonic memory applications proposed below, where the damage due to cycling should not occur. Bulk and nanostructured VO_2_ films are well-known to withstand millions of electrical or optical switching cycles.^46^

In the previous sections, we showed that the change in ADF intensity during the phase transition is related to the change in the EEL spectrum and that the EEL properties can be directly linked to optical properties (see Figure S3b). This means that ADF levels observed in Figure 4c can be considered as four optical transmission levels, e.g., when NPs are placed on top of a waveguide. Such an ensemble of *n* NPs thus represents a mesoscale version of the memory unit described at the beginning of this section and shown in Figure 4a, where the memory levels scale as *n* + 1. Because the MIT of these NPs occurred below 40 °C, the retention energy of such memories is significantly lower than that of the current VO_2_ optical memories. However, for larger NPs, where the transmission contrast is larger (as shown in Figure S3a), the hysteresis is narrower and thus offers less room for memory stability. As there might be a certain minimal required transmission change for a practical memory application, this contrast–width trade-off must be carefully considered during the design process. Another consideration must be taken when choosing and addressing NPs due to the statistical distribution of the IMT temperatures, which significantly lowers the number of possible levels, especially when this number depends on the NP size and NPs are switched simultaneously. The ultimate limit of available levels is set by the number of NPs within the area available for the mesoscale memory unit. It is true, however, that the stochastic nature of the transitions (mostly that of IMT) might further reduce it, especially when a large number of levels/NPs will be required. This requirement will naturally arise from the specific application. In the most realistic case where the memory levels would be controlled via different transition temperatures tied to the size of the NP, the stochasticity of the IMT interval of a single NP around ±2 °C and the achievable range of transition temperatures bounded between 67–90 °C, the maximum number of levels will be realistically limited to the order of ten or low tens. We envision that if the phase transition control over the VO_2_ NPs is improved in the future to a single-NP limit, the number of levels can indeed be limited to the number of NPs. This can be achieved, for example, by direct electrical contacts or by optical control using spatial light modulators. Nevertheless, the demonstrated VO_2_ NP memory, which provides several levels of optical transmission with a submicron footprint accessible by ultrafast electrical or optical pulses with low peak powers, proves to be another noteworthy candidate for optical memories used in future data storage and processing.

## Conclusions

In conclusion, we have studied individual single-crystal VO_2_ NPs with STEM techniques and showed that their plasmonic fingerprints, appearing in the EEL spectra of NPs in the metallic phase, serve as a reliable high-contrast tool for tracking phase transitions. By correlating EEL measurements with far-field optical properties, we proved that larger NPs exhibit greater optical (absorption) contrast in the telecom region upon the transition. Then, we dynamically recorded the evolution of EEL spectra and ADF images during the respective heating and cooling cycles. We obtained hystereses of individual NPs, verified the coexistence of the insulator and metal phases during the IMT and proved that the intensity of ADF images of NPs can be used to correctly recreate the hysteresis obtained by the EEL measurement. While the electron beam was found to gradually lower the MIT temperature and therefore impact the measurement, analyzing the transition using ADF images is a 100-times less-exposing and three times faster technique than the EEL measurement. The ADF videos allowed us to simultaneously study 266 NPs and obtain their IMT and MIT temperatures and transition widths. We found that with the increasing NP volume, the MIT temperature also increases, and thus, the hysteresis becomes narrower, while the statistical distribution of the MIT temperatures is more than two times larger than that of the IMT. Based on these results and the evidence that the MIT is more than three times sharper than the IMT (coexisting phases 70 times less probable), we proposed a model for the overall hysteresis of a polycrystalline VO_2_ film. In the model, the hysteresis is formed by IMTs of individual NPs, whose temperatures are spread narrowly but transition widths are broad, and by MITs, whose temperatures are distributed across a larger temperature window, but the transitions are extremely abrupt. Lastly, we showed that the coexisting phases of the individual NPs are not persistent at lower temperatures, and therefore, the individual NP cannot be used as a long-term multilevel memory kept at near room temperature. This finding proved that the intermediate states (levels) presented in the macroscale transmission memory made of an ensemble of VO_2_ NPs represent a “digital” mixture of either fully switched or nonswitched NPs. Finally, we established a concept of mesoscale memory working at mere 40 °C, where the number of levels scales with the number of NPs as *n* + 1. Despite the discussed contrast–width trade-off (larger NPs have more considerable transmission contrast but higher MIT temperature; larger retention temperature is needed), such VO_2_ NP optical memory with already several levels of information at the submicron footprint can surpass current VO_2_ optical memory patches. While unsuitable for long-term data storage due to the requirement for constant energy supply,^3^ this low-temperature hysteresis with ultrafast transition makes VO_2_ one of the candidates for optical random access memories^8^ or for optical neuromorphic computing that often require only short-term data retention.^7^

## Methods

### Fabrication

VO_2_ NPs on fused silica and SiN heating chip membrane (Protochips Inc., USA) were fabricated in a two-step process. First, the 30 nm amorphous film was fabricated by TSST pulsed laser deposition (PLD) system (248 nm KrF laser, 2 J/cm^2^, 10 Hz, 50,000 pulses, vanadium target (99.9% purity, Mateck GmbH), 50 mm substrate–target distance, room temperature and 5 mTorr oxygen pressure) and then annealed *ex-situ* for 30 min in a vacuum furnace (Clasic CZ Ltd.) at 700 °C and 15 sccm oxygen flow.

### Characterization

Transmission and ellipsometry of the VO_2_ NPs on fused silica and VO_2_ film on silicon, respectively, were carried out using a spectroscopic ellipsometer J. A. Woollam V–VASE with an adjustable compensator, 1000 μm monochromator slit, 50, 60, and 70° incident angles (for ellipsometry), 3 mm spot size and 0.5–4 eV spectral range with 0.04 eV step. The extinction was calculated as 1 – *T*, where *T* is the transmission. The temperature was feedback-controlled during the spectroscopic measurements by a home-built heating stage, which included a resistive heater and a thermocouple.

Scanning transmission electron microscopy with EEL spectroscopy was performed using TEM FEI Titan equipped with a monochromator, GIF Quantum spectrometer for EELS, and *in situ* Fusion Select system by Protochips for heating experiments. We used the following parameters: primary beam energy of 120 keV, electron beam current around 100 pA, convergence semiangle of 8.14 mrad, ADF collection angle 16.7–38.3 mrad and exposure time 1–2 μs/px, and EELS collection angle 8.3 mrad and exposure time 0.2–0.3 ms/px. EEL spectra were integrated over marked regions of interest and further processed by subtracting a reference EEL spectrum integrated over the pure SiN membrane (i.e., an area far away from the VO_2_ NP within the respect spectrum image). The spectra were then normalized with respect to integral zero-loss peak intensity (energy window from −1 to +1 eV); to transform counts to a quantity proportional to the EEL probability and divided by the spectrometer dispersion (0.01 eV/px; to obtain loss probability density in units eV^–1^ referred to as the EEL probability).

Note that the elevated temperature during the fabrication process corrupted the heating element on the membrane, so the heating chip temperatures had to be recalibrated (see Figure S8).

### Processing of ADF Intensity for Hysteresis and Statistics

After recording the ADF video during the phase transition of VO_2_ NPs using Protochips Inc. software Axon, we extracted a sequence of images and processed them using the ImageJ software^47^ and StackReg plugin.^48^ Specifically, we aligned images using the StackReg plugin, created a mask around each of the NPs, and measured the mean gray value of masked NPs simultaneously across all images/temperatures. The transition temperatures and transition widths of each NP were obtained from the position and full width at half-maximum (FWHM) of a Gaussian profile fitted to the first-order derivative of either the IMT or MIT parts of the hysteresis curve. Transition widths were calculated as 6 standard deviations of the fitted normal Gaussian distribution, which equals 2.55 FWHM. This value is equivalent to the temperature range it takes to switch fully from one phase to another in a 99.8% confidence interval. In Figure 4c, the ADF intensities of NPs were summed (or subtracted in the case of the first switched NP) for a clear demonstration of the memory effect. In the case of an actual optical device, the transmitted intensity is less noisy and always drops during the IMT as the light absorption of the NP in the metallic phase is larger.

### Simulation

Extinction of hemispherical VO_2_ NP arrays on fused silica and extinction coefficient of single hemispherical VO_2_ NPs in air were calculated using the finite-difference time-domain (FDTD) method implemented in the Ansys Lumerical FDTD Solutions software. The distance between the NPs and the horizontal FDTD region boundary was always kept at least half of the longest recorded wavelength. Conformal meshing (mesh order 4) was adopted everywhere except the NPs, where we set the staircase meshing with a 5 nm step. We employed a total-field scattered-field (TFSF) source with 16 layers of perfectly matched layers boundary conditions for individual NP simulations and a plane wave source with periodic boundary conditions and double-diameter square spacing for the simulations of the arrays. The NPs were illuminated from the bottom/substrate. The extinction of arrays was calculated as 1 – *T*, where *T* is the transmission, which was obtained from the monitor above the NPs. The extinction coefficient of individual NPs was obtained by dividing the sum of scattering and absorption cross sections by the diameter of the NP. All simulated and experimental data in Figure S3 were normalized to the maximum value at the largest investigated 130 nm NP to compare the trend of their properties.

EEL spectra of hemispherical VO_2_ particles of various radii were calculated using the commercial software COMSOL Multiphysics, utilizing classical dielectric formalism,^49^ and following the procedure reported previously.^50^ We assumed the nonrecoil approximation, where the electron trajectory is approximated by a straight line element parallel with the *z* direction, along which we set the current *I* = *I*_0_e^i*ωz*/*v*^, where *I*_0_ is the current amplitude, ℏω is the energy loss with ℏ the reduced Planck constant, and *v* is the electron velocity (set to 0.587 of speed of light to match the acceleration voltage 120 keV). The EEL probability can be expressed as
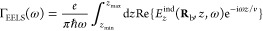
1where *E*_*z*_^ind^ is the *z*-component of the induced electric field, **R**_b_ = (*x*_b_, *y*_b_) is the electron beam position relative to the center of the NP, located in the origin, and *e* is the elementary charge. We integrate over electron trajectory restricted by the simulation domain *z* = (*z*_min_, *z*_max_). The boundary conditions in the form of perfectly matched layers were imposed, and the simulation domain was kept sufficiently large (always larger than the wavelength considered). The simulation mesh was refined inside the NPs (max size 7.5 nm) and coarser for the surrounding domain, for which we considered ϵ_r_ = 1.

The material response of all simulated NPs is characterized by the dielectric function (Figure S9) obtained by spectroscopic ellipsometry from the VO_2_ thin film deposited by PLD and annealed for 10 min at 600 °C and 15 sccm oxygen flow.

## Supporting Information Available

Video 1: Sequences of STEM ADF images capturing the phase transition hystereses of hundreds of VO_2_ NPs (AVI)
Video 2: Sequences of STEM ADF images capturing cumulative switching of VO_2_ NPs during three heating–cooling cycles (AVI)
Shape and size distribution of VO_2_ NPs; Decomposition of the plasmonic peak; Correlation of EEL spectra and far-field optical properties; EEL spectra of coexisting phases within one NP; Metallic phase conservation by the electron beam; Coexisting phases during the MIT; Decreasing phase transition temperatures with electron beam illumination; SiN heating chip temperature calibration; Dielectric function of VO_2_ thin film (PDF)

## Supplementary Material

nn4c13188_si_001.avi

nn4c13188_si_002.pdf

nn4c13188_si_003.avi
