# Supplementary material for: Coexisting Phases of Individual VO2 Nanoparticles for Multilevel Nanoscale Memory
Source: ACS Nano. 2025 Jan 2;19(1):1167–76. doi: 10.1021/acsnano.4c13188 (PMC11752518; doi:10.1021/acsnano.4c13188)
Supplement: Supplementary file 2 — nn4c13188_si_002.pdf [file nn4c13188_si_002.pdf]

Supporting Information: Coexisting phases of individual VO_2 nanoparticles for multilevel nanoscale memory

Peter Kepič,[*,†] Michal Horák,[†] Jiří Kabát,[†,‡] Martin Hájek,[‡] Andrea Konečná,[†,‡] Tomáš Šikola,[†,‡] and Filip Ligmajer[*,†,‡]

†*Brno University of Technology, Central European Institute of Technology, Purkyňova 123, 612 00, Brno, Czech Republic*

‡*Brno University of Technology, Faculty of Mechanical Engineering, Institute of Physical Engineering, Technická 2, 616 69, Brno, Czech Republic*

E-mail: peter.kepic@ceitec.vutbr.cz; filip.ligmajer@ceitec.vutbr.cz

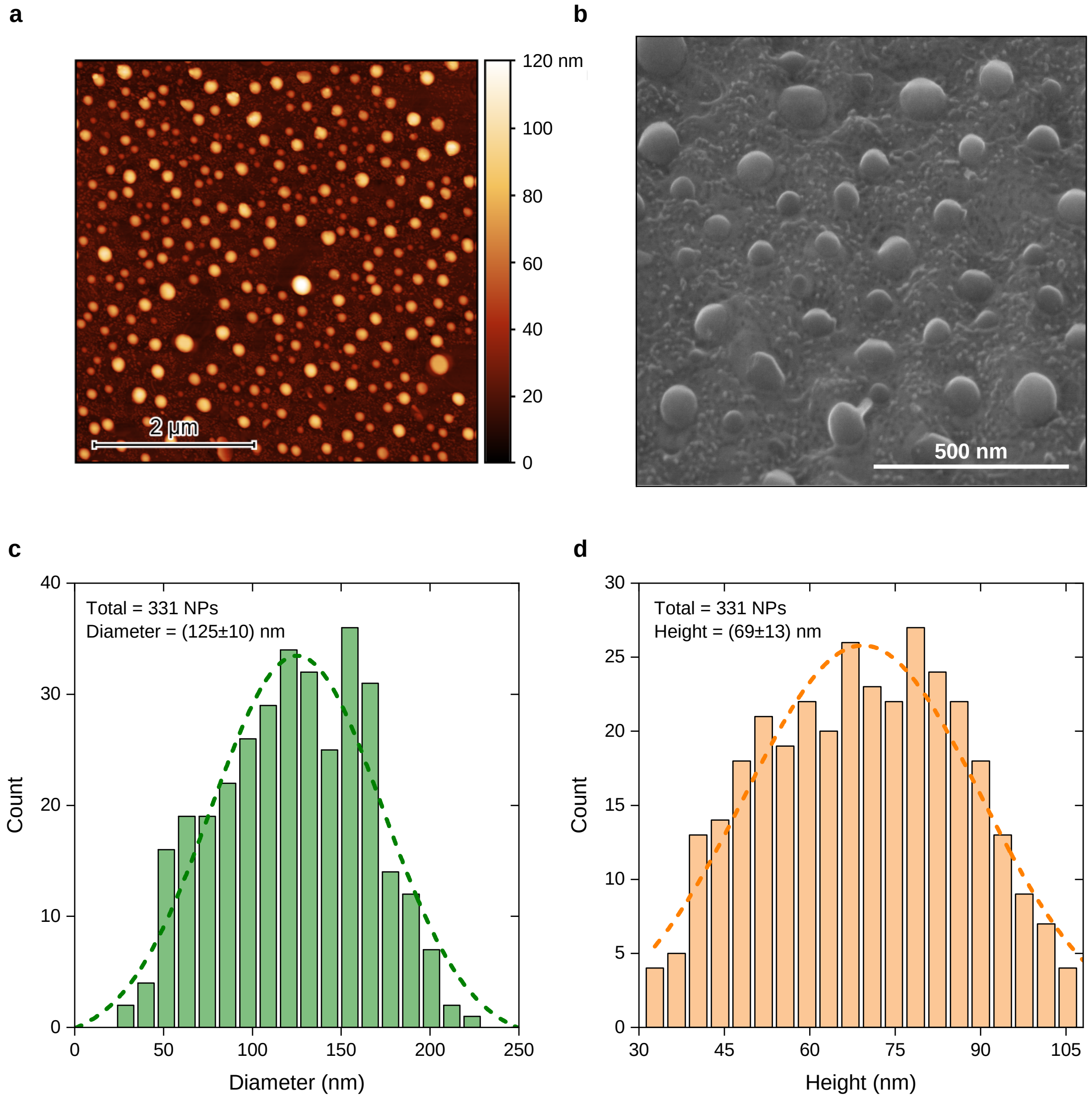

Fig. S1: Shape and size distribution of VO_2 NPs. (a) AFM image and (b) SEM micrograph of the VO_2 NPs on the fused silica and SiN heating chip membrane, respectively. (c) Diameter and (d) height distribution of VO_2 NPs from the AFM image in (a).

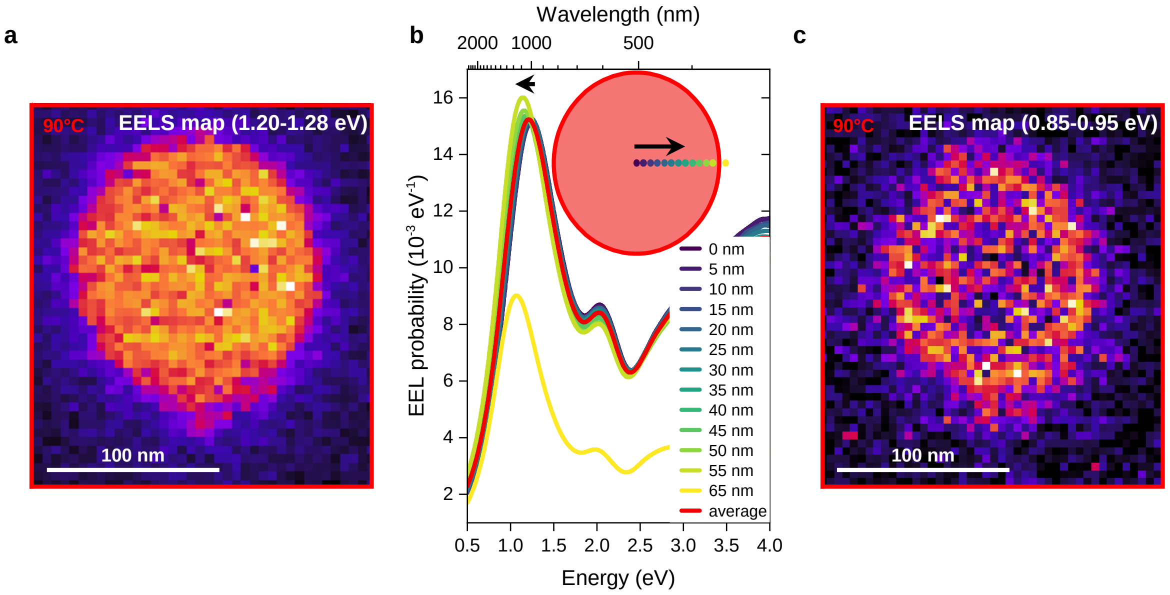

Fig. S2: Decomposition of the plasmonic peak. (a) EEL intensity map of the investigated NP in Figure 1c in the metallic phase, summed between 1.20–1.28 eV. (b) Simulated EEL spectra of the metallic spherical VO_2 NP with the 130 nm diameter in vacuum, illuminated by an electron beam in the listed positions that are also highlighted on the scheme of the NP. (c) EEL intensity map of the investigated NP in Figure 1c in the metallic phase, summed between 0.85–0.95 eV. The plasmonic peak in Figure 1c is formed by two contributions: Predominantly by the volume plasmon, with the homogeneous spatial distribution of the loss probability in (a), and partially by the localized surface plasmon resonance around 1 eV, with the EEL probability having a maximum near the boundary of the NP, as shown in (b) and (c).

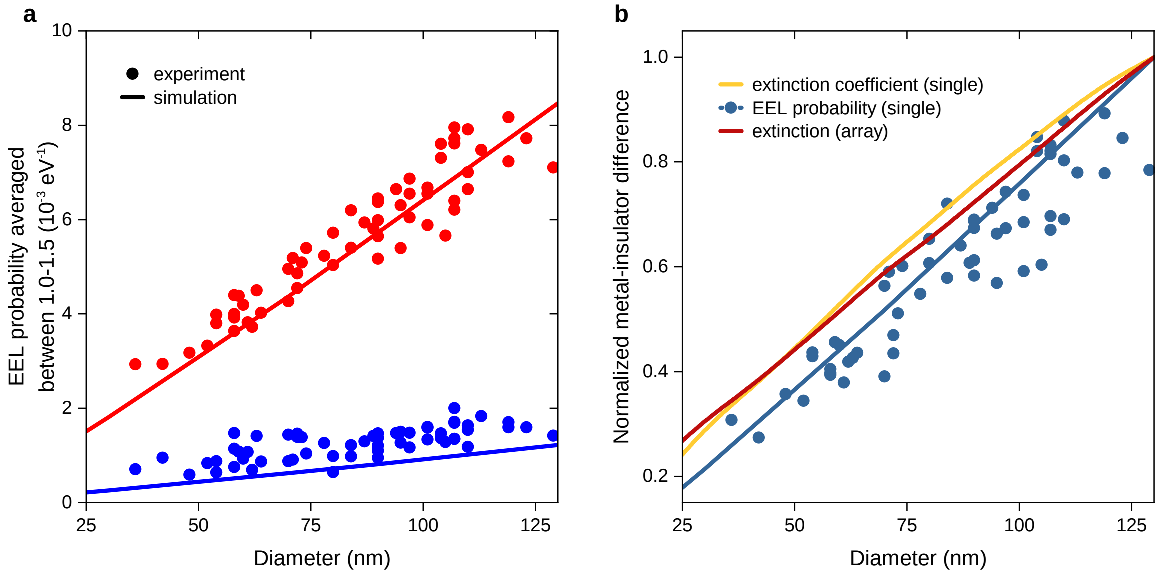

Fig. S3: Correlation of EEL spectra and far-field optical properties. (a) Experimental and simulated averaged EEL probability of various VO_2 NPs as a function of their diameter at the insulator (blue) and metal (red) phase. The averaged EEL probability represents the spatially integrated EEL probability over each NP, and calculated the average value in the region around the resonance between 1.0–1.5 eV. (b) Comparison of the simulated optical extinction coefficients and EEL intensities of a single NP and of a periodic array of NPs and experimental EEL intensities in the form of normalized metal-insulator differences of the respective spectra as a function of the NP diameter. Simulated switching contrasts were extracted in the same energy window as in the experiment and normalized to unity. As the calculated optical switching contrasts correspond well to the EEL spectroscopy measurements and all results exhibit approximately a linear dependence on the NP size, we can relate EEL measurements to far-field optical properties and utilize them to investigate possible applications in memories or metasurfaces.

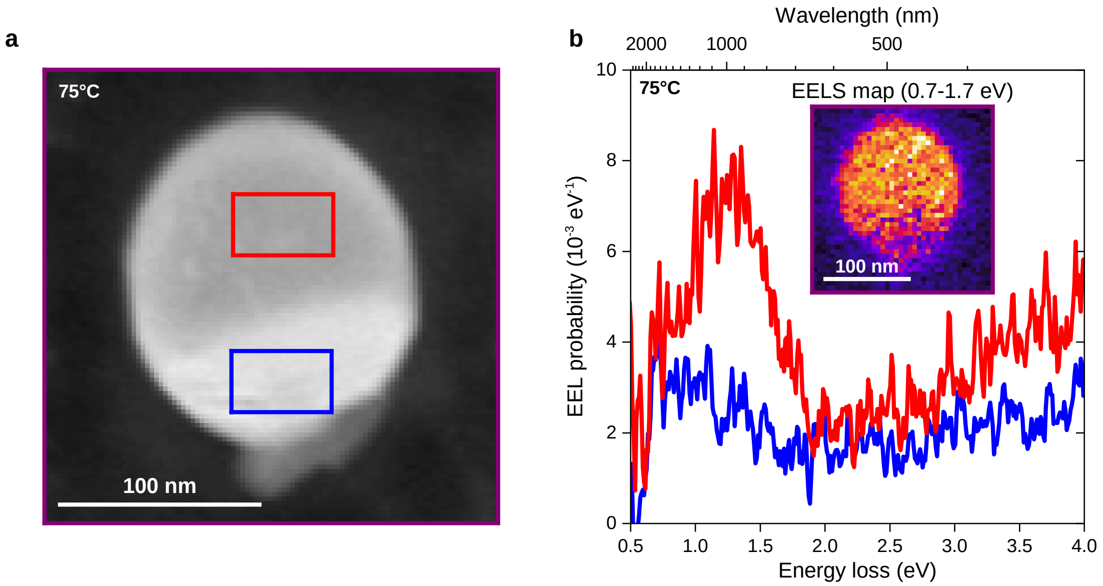

Fig. S4: EEL spectra of coexisting phases within one NP. (a) ADF image and (b) EEL spectra of two coexisting phases within the single VO_2 NP recorded at 75 °C. The inset shows an EEL intensity map of the NP integrated between 0.7–1.7 eV. The blue and red rectangles in (a) highlight the areas from which the spectra were averaged. This figure shows that while the EEL spectrum from the red area implies this region is metallic, the spectrum from the blue area indicates the coexisting insulating phase in that region. Correspondingly, the EEL intensity map of the NP integrated between 0.7–1.7 eV shows low intensity at the bottom of the particle, where this insulating phase is located.

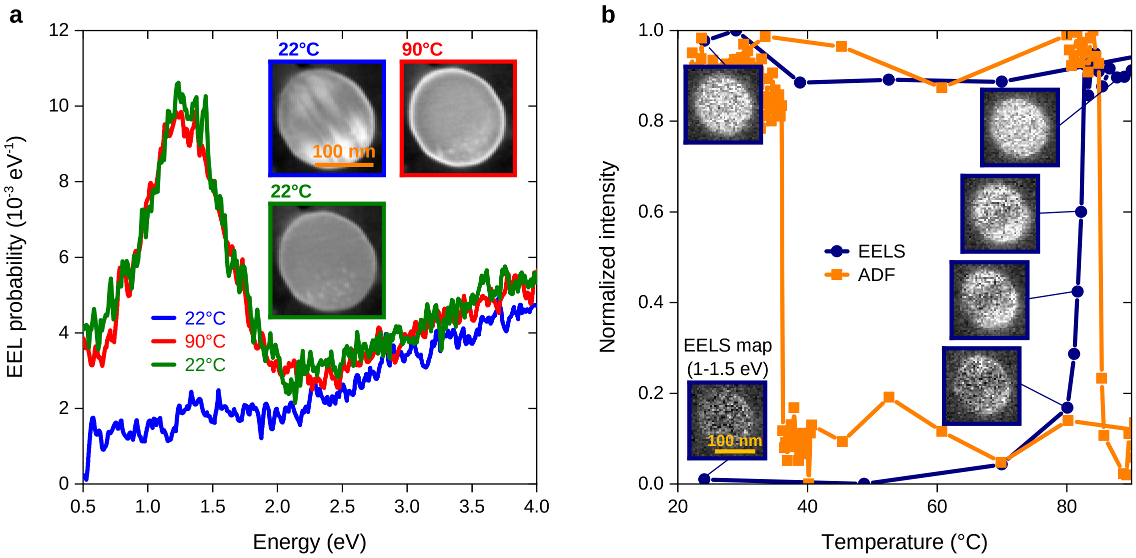

Fig. S5: Metallic phase conservation by the electron beam. (a) Measured EEL spectra of the VO_2 NP with the 150 nm diameter obtained at 22 °C (blue), 90 °C (red) and again 22 °C (light blue). Exhibiting the plasmon resonance, the NP remained in the metallic state after cooling back to room temperature. The inset shows ADF images at the listed temperatures. (b) Phase transition hysteresis of the normalized EEL intensity averaged between 1.0–1.5 eV and ADF intensity of the NP in (a). The insets show the EEL intensity maps of the NP integrated between 1.0–1.5 eV and measured at the marked temperature positions. The conservation effect was observed for several NPs when illuminated after a certain electron dose. To understand the effect and determine specific doses, a study that is beyond the scope of this paper has to be carried out.

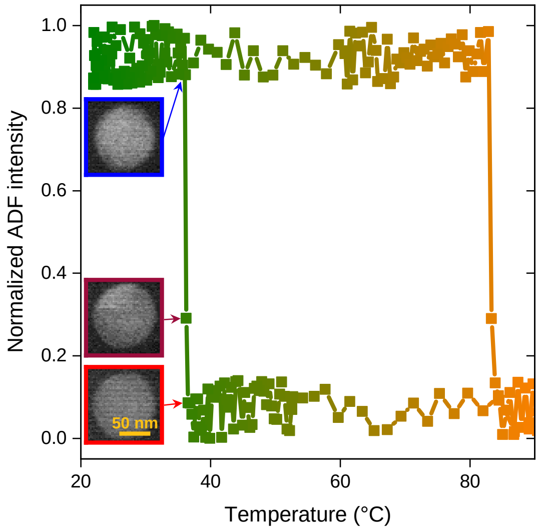

Fig. S6: Coexisting phases during the MIT. The phase transition ADF intensity hysteresis of the VO_2 NP with the 100 nm diameter, extracted from the ADF images. The insets show ADF images during the MIT. The NPs that exhibited coexisting phases during both transitions represent approx. 1 % of the studied NPs.

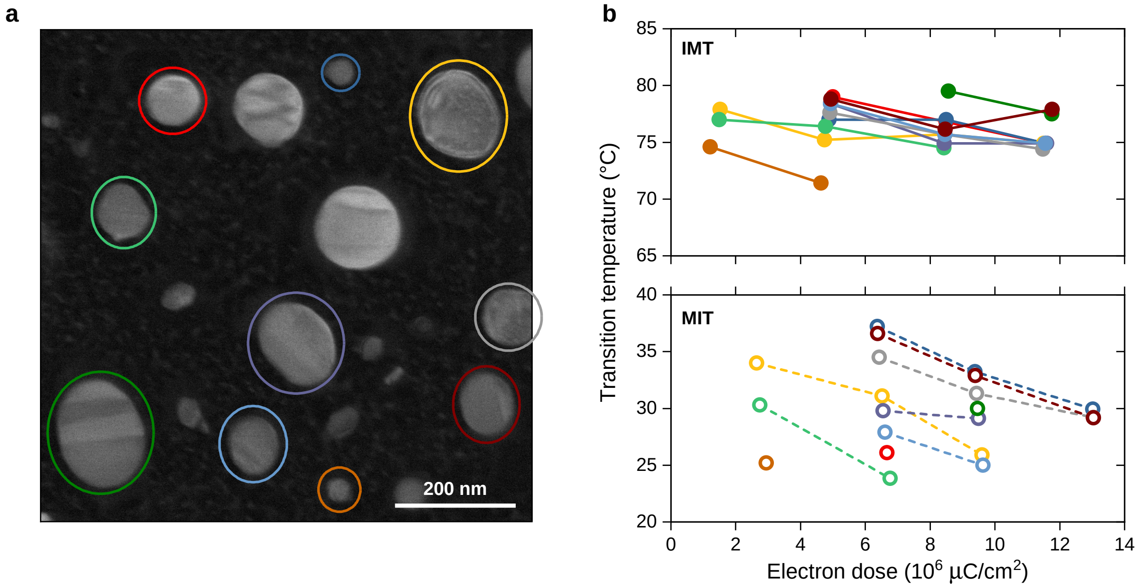

Fig. S7: Decreasing phase transition temperatures with electron beam illumination. (a) The ADF image of the VO_2 NP area that underwent four heating–cooling cycles and the area from which 3 NPs for mesoscale memory were chosen. The full cycling can be seen in Supporting Video 2. (b) IMT and MIT temperatures of highlighted NPs in (a) as functions of the electron beam dose. The graph shows the decreasing trend of transition temperatures with the increasing electron dose, where the decrease of the MIT temperature is more pronounced. The electron dose was calculated by multiplying the number of the images taken up to the specific transition temperature with an estimated dose 1.2×10^4 $\mu C/cm^2$ per image. Therefore, data points are shifted to the right when IMT temperatures during heating are increasing, and MIT temperatures during cooling are decreasing. Data points are connected with lines to guide the eye.

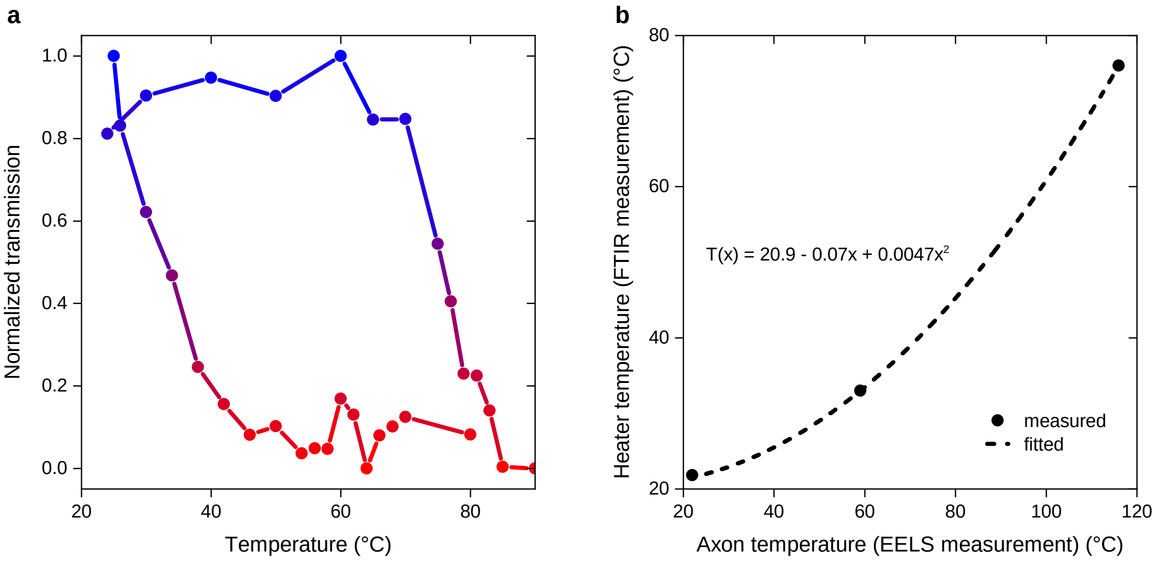

Fig. S8: SiN heating chip temperature calibration. (a) Phase transition hysteresis of normalized transmission in the near-infrared of VO_2 NPs on the SiN heating chip membrane. (b) The calibration curve between the temperature displayed in Protochips Inc. software Axon and the temperature displayed by the trusted ex-situ heater. X-coordinates of data points were obtained as the average transition temperatures of hundreds of NPs, processed as in Figure 3. Y-coordinates of data points were obtained from the transition temperatures in (a), measured by the ex-situ Fourier transform infrared spectroscopy with the home-built heating stage. The first data point represents an equal room-temperature starting point. Data were fitted by an empirical quadratic function displayed in the graph.

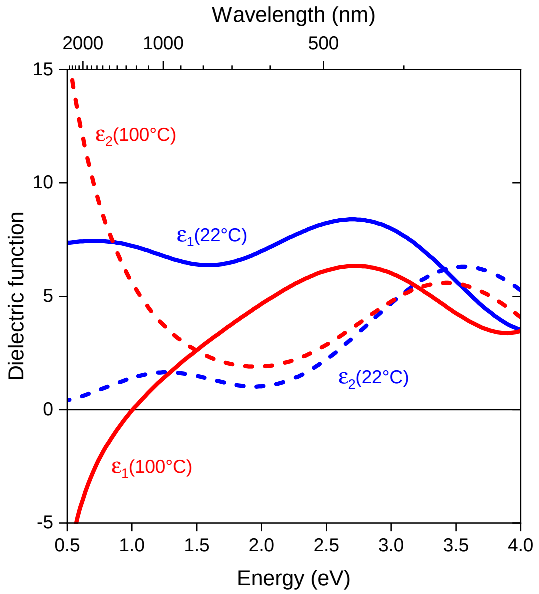

Fig. S9: Dielectric function of VO_2 thin film. Real (full line) and imaginary (dashed line) parts of the dielectric function of a 30 nm VO_2 thin film on the silicon substrate obtained at 22 °C (blue) and 100 °C (red) in the insulator and metallic phases, respectively.
